# Discontinuity Characterization and Low-Complexity Smoothing in RF-PA Polynomial Piecewise Modeling

**DOI:** 10.3390/s25216593

**Published:** 2025-10-26

**Authors:** Carolina Pedrosa, Dang-Kièn Germain Pham, Peter Rashev, Pierre Almairac, Jean-Christophe Nanan, Patricia Desgreys

**Affiliations:** 1 Laboratoire Traitement et Communication de l’Information (LTCI), Télécom Paris, Institut Polytechnique de Paris, 91120 Palaiseau, Francepatricia.desgreys@telecom-paris.fr (P.D.); 2 NXP Semiconductors, 134 Av. du Général Eisenhower, 31100 Toulouse, France; 3 NXP Semiconductors, 1 Hines Rd., Kanata, ON K2K 3C7, Canada

**Keywords:** digital predistortion, generalized memory polynomial, piecewise modeling, power amplifier (PA), 5G, continuity

## Abstract

Piecewise modeling of power amplifiers (PAs) typically involves assembling different polynomials to capture nonlinear behavior across different operating regions. However, recombining these sub-models can introduce discontinuities at segment boundaries, degrading prediction accuracy and potentially impacting digital predistortion (DPD) performance. This work addresses this issue by introducing a statistical framework to detect discontinuities through localized variations in the conditional mean and variance of amplitude and phase responses. Using the Vector-Switched Generalized Memory Polynomial (VS-GMP) as a case study, we propose a low-complexity post-processing smoothing technique based on a raised cosine weighting function applied at model transition regions. Unlike structural approaches, the method requires no retraining and integrates seamlessly into existing workflows as a post-processing tool. Experimental validation across two PA architectures (Doherty and Single-Stage) and multiple 5G/LTE signals (20–200 MHz bandwidth, up to 11 dB PAPR, including carrier aggregation) demonstrates consistent improvements: up to a 3 dB NMSE reduction and notable spectral error suppression.

## 1. Introduction

Wireless sensor systems, such as IoT and remote monitoring networks, depend on power-efficient and linear RF transmission to ensure reliable data delivery. Nonlinear distortion introduced by power amplifiers degrades spectral efficiency, increases interference, and can compromise the integrity of sensor data links.

Piecewise modeling of power amplifiers (PAs) [1] is a common approach to capturing their nonlinear behavior across different operating regions. Global polynomial models often underperform in situations where the amplitude distortion is sinuous, such as PAs with multiple amplification stages [2,3] in deep compression.

Methods such as Vector-Switched Models [4,5] and Machine Learning Classification [6,7] will decompose the dataset into different sub-models to reduce the overall system complexity without compromising accuracy.

While this decomposition improves modeling accuracy, it introduces a new challenge. Recombining different models into a single cohesive dataset can introduce discontinuities at the boundaries between the piecewise segments, leading to inaccurate representations of the PA response. These effects are exacerbated when very different polynomial structures are used in each of the partitions. The discontinuities can cause numerical instability in simulations, degrade the performance of digital predistortion (DPD) algorithms, and introduce spectral artifacts in communication systems.

Although discontinuities in piecewise models have been studied in control systems and fuzzy modeling, their implications for RF power amplifier (PA) modeling—particularly with polynomial-based approaches—are still not frequently discussed. This gap is especially relevant given that most digital predistortion (DPD) techniques currently used in base station transmitters rely on polynomial behavioral models. Johansen and Foss [8] addressed discontinuities in NARMAX models through regime decomposition and smooth weighting functions. Similarly, Takagi and Sugeno [9] proposed fuzzy identification methods using bell-shaped membership functions to ensure smooth transitions between regions. In the context of LUT-based PA modeling, Barradas et al. [10] showed that spline-interpolated LUTs and smooth basis functions can help maintain continuity across segments, and Zhijian Yu [11] evaluates interpolation techniques using the Farrow structure for local support piecewise DPD; however discontinuities in polynomial-based piecewise models arise from differences in polynomial coefficients across regions, not from missing intermediate points, as is often the case for LUTs. Therefore, these methods do not apply directly in the context of polynomial-based piecewise modeling and cannot be used for comparison.

In the context of smoothed polynomial piecewise modeling, Brihuega et al. [12] introduce a Mixture of Experts (ME) framework that inherently ensures continuity through soft partitions and probabilistic gating. However, these methods often require structural changes to the model or retraining with overlapping basis functions, which is often impractical in commercial environments. In contrast, our approach applies smoothing as a post-processing step, preserving the original model coefficients and enabling modular integration into existing workflows. This distinction makes our method particularly attractive for practical deployment in RF PA modeling pipelines. Furthermore, unlike prior works that qualitatively address discontinuities through architectural design, our method introduces a statistical framework to quantitatively characterize discontinuities using the conditional mean and variance. To the best of our knowledge, this form of statistical discontinuity assessment has not been previously applied in the context of RF PA behavioral modeling, offering a new lens to evaluate and mitigate model inconsistencies.

The methodology of this work is divided into two main parts. First, a statistical approach is introduced to detect discontinuities in piecewise models by analyzing the variation in the mean and spread of the predicted amplitude and phase responses near the segment boundaries. This method helps identify where the sub-models do not align smoothly. Then, a smoothing technique is applied using a raised cosine weighting function to blend the outputs of adjacent sub-models in the overlapping regions. This smoothing is performed during post-processing, without changing the original model coefficients. The goal is to improve the continuity of the predicted signal and reduce modeling artifacts, while keeping the implementation simple and compatible with existing modeling workflows.

## 2. Theoretical Description of Smoothed Segmented Models

### 2.1. Piecewise Polynomial Modeling

#### 2.1.1. Standard Approach

Piecewise polynomial models are functions defined over a domain partitioned into segments, where a distinct polynomial governs the behavior in each segment. Let the power domain [a,b]⊂R be divided into *N* subintervals by a sequence of breakpoints (also known as knots) $a=x_{0}<x_{1}<\cdots <x_{N}=b$. On each interval $\left[x_{i-1},x_{i}\right)$, the data is modeled by a polynomial $P_{i}\left(x\right)$ of degree $d_{i}$:

(1) $$f\left(x\right)=P_{i}\left(x\right)=\sum_{p=0}^{d_{i}}a_{i,p}x^{p},\;\;\mathrm{for}\;x\in I_{i}=\left[x_{i-1},x_{i}\right).$$

For simplicity, we consider the power domain to be real-valued. However, these formulations can be generalized to the complex domain to accommodate more general signal representations.

For clarity, we introduce rectangular functions (also known as indicator or box functions), which isolate each segment:

(2) $${\mathrm{rect}}_{i}\left(x\right)=\left\{\begin{array}{cc} 1 & \mathrm{if}\;x\in I_{i}=\left[x_{i-1},x_{i}\right)\\ 0 & \mathrm{otherwise}. \end{array}\right.$$

All rectangular functions are orthogonal to each other (non-overlapping) and

(3) $$\sum_{i=1}^{N}{\mathrm{rect}}_{i}\left(x\right)=1,\;\forall x\in \left[a,b\right].$$

Using the rectangular functions, the full piecewise model can be expressed as a single sum:

(4) $$f\left(x\right)=\sum_{i=1}^{N}P_{i}\left(x\right)\cdot {\mathrm{rect}}_{i}\left(x\right).$$

**Figure 1:**
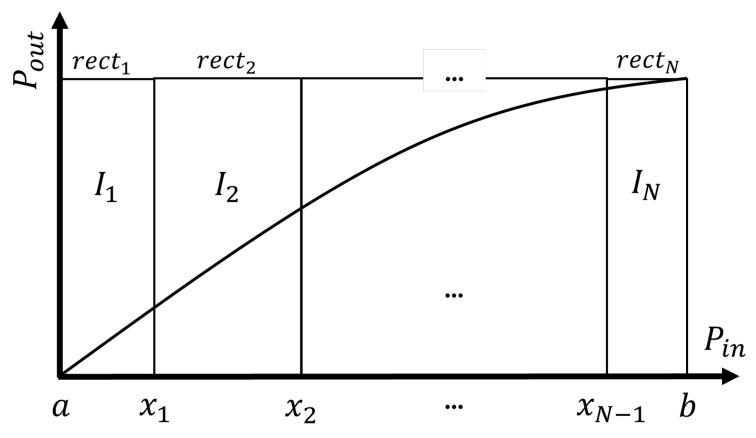
Diagram of the standard approach to piecewise modeling.

The conventional piecewise polynomial modeling approach is to use Equation (4) for both generating the models (identification) and predicting the outputs (inference). The diagram in Figure 1 shows the partitioning of the data into rectangular power regions.

This formulation is both straightforward to implement and easy to interpret. However, it naturally introduces discontinuities at the segment boundaries. Such discontinuities may arise from the classical polynomial non-convergence effect known as Runge’s phenomenon [13] or, more critically, when the polynomial composition—such as the kernel type (e.g., MP, GMP, and Volterra) or model complexity—differs significantly between adjacent intervals. To address these limitations, we propose the use of overlapping basis functions, which provide a smoother transition across segments and mitigate abrupt changes in model behavior.

#### 2.1.2. Overlapping Basis Functions

**Figure 2:**
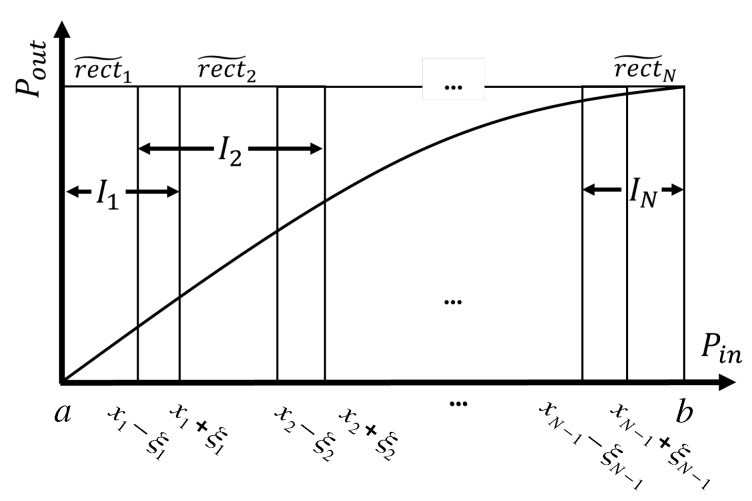
Diagram of the modified rectangular function with overlapping intervals.

To mitigate discontinuities, we consider the case where the orthogonal rectangular functions are replaced with overlapping functions (also called blending functions), as depicted in Figure 2.

The associated intervals are defined as extended versions of the initial intervals:

(5) $$I_{i}=\left[x_{i-1},x_{i}\right)\to \tilde{I_{i}}=\left[x_{i-1}-\xi_{i-1},x_{i}+\xi_{i}\right)$$

where $\xi_{i}$ is related to the overlap width; please note that for a given $x_{i}$, intervals overlap on $\left[x_{i}-\xi_{i},x_{i}+\xi_{i}\right]$. Though it is not mandatory, we set $\xi_{i-1}=\xi_{i}$ for simplicity. Now data in each interval $\tilde{I_{i}}$ is modeled by:

(6) $$\tilde{P_{i}}\left(x\right)\cdot {\tilde{\mathrm{rect}}}_{i}\left(x\right),$$

where $\tilde{P_{i}}\left(x\right)$ is the new polynomial of degree $d_{i}$ that fits the data on $\tilde{I_{i}}$ and

(7) $${\tilde{\mathrm{rect}}}_{i}\left(x\right)=\left\{\begin{array}{cc} 1 & \mathrm{if}\;x\in \tilde{I_{i}}=\left[x_{i-1}-\xi_{i-1},x_{i}+\xi_{i}\right)\\ 0 & \mathrm{otherwise}. \end{array}\right.$$

Note that the polynomials $\tilde{P_{i}}\left(x\right)$ are actually identified on the extended intervals $\tilde{I_{i}}$, which leads to different coefficients compared to $P_{i}\left(x\right)$ identified on $I_{i}$.

The full piecewise model can then be expressed as follows:

(8) $$f\left(x\right)=\sum_{i=1}^{N}\tilde{P_{i}}\left(x\right)\cdot {\mathrm{rect}}_{i}\left(x\right).$$

Even though this approach helps minimize the appearance of discontinuities at the knots, it will also discard the overlap data at the inference phase. To maximize the usage of all available data, the proposed solution uses overlapping basis functions denoted as ${\tilde{\phi }}_{i}\left(x\right)$.

Please note that $\sum_{i=1}^{N}\tilde{P_{i}}\left(x\right)\cdot {\tilde{\mathrm{rect}}}_{i}\left(x\right)$ is fundamentally wrong because the overlapping rectangular functions are cumulating in the overlapping regions. More precisely, $\tilde{P_{i}}\left(x\right)$ will cumulate with $\tilde{P_{i+1}}\left(x\right)$ in the overlapping region $\left[x_{i}-\xi_{i+1},x_{i}+\xi_{i}\right]$ and the same for $\tilde{P_{i-1}}\left(x\right)$ in the overlapping region $\left[x_{i-1}-\xi_{i},x_{i-1}+\xi_{i}\right]$.

**Figure 3:**
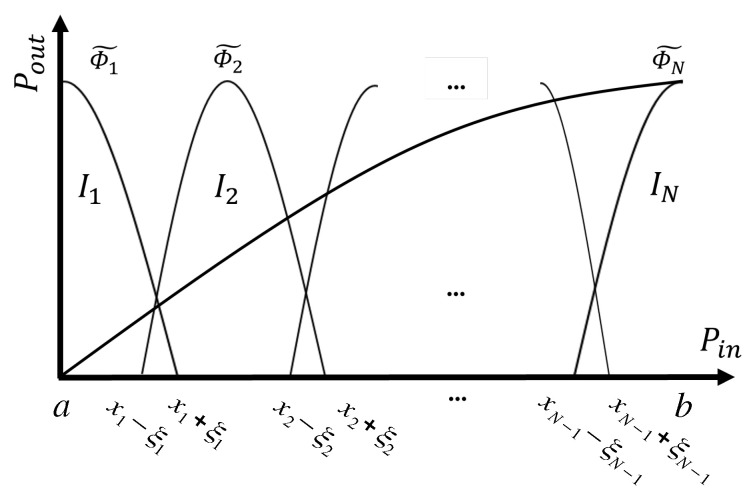
Overlapping basis functions approach to piecewise modeling.

A natural choice for overlapping basis functions is to use functions that are continuous and gradually transition from 0 to 1 along the overlapping range (around the knots). These functions blend the data in the transition regions, as depicted in Figure 3. It is important to note that these functions are not orthogonal to each other; however, they are chosen so as to satisfy

(9) $$\sum_{i=1}^{N}{\tilde{\phi }}_{i}\left(x\right)=1,\;\forall x\in \left[a,b\right]$$

To conclude, we consider the following blended piecewise polynomial model:

(10) $$f\left(x\right)=\sum_{i=1}^{N}\tilde{P_{i}}\left(x\right)\cdot {\tilde{\phi }}_{i}\left(x\right).$$

Equation (10) is generalized to complex signals by using

(11) $$f\left(x\right)=\sum_{i=1}^{N}\tilde{P_{i}}\left(x\right)\cdot {\tilde{\phi }}_{i}\left(|x|\right)$$

where x∈C, $\tilde{P_{i}}$ is a complex-valued polynomial, and |x| is the modulus of *x*. Given Equation (11), the model can be identified using two approaches:

- Overlap-aware approach: The model is identified using a least-squares method and the blended polynomial model in Equation (10) over the entire range [a,b].
- Orthogonal interval approach: The model is identified using a least-squares method for each interval independently solving $\tilde{P_{i}}\left(x\right)\cdot {\tilde{\mathrm{rect}}}_{i}\left(x\right)$, and blending functions are then used to smooth the transitions between the segments using Equation (10) at the inference (post-processing) phase.

The overlap-aware approach ensures continuity is well-established in system identification and fuzzy modeling. All the cited works—Johansen and Foss [8,14], Takagi and Sugeno [9], Barradas et al. [10], and Brihuega et al. [12]—implement smoothing directly within the modeling framework through overlapping partitions, soft gating, or smooth basis functions. None of these approaches rely on post-processing to enforce continuity, as we propose in this work.

The overlap-aware approach is expected to provide the best fit; however, it is also the most complex one: new equations have to be added to the optimization problem, which can be difficult to implement in very optimized/specialized coding environments (like in large commercial tools).

Our work builds on these foundations but introduces a key practical innovation: smoothing is only applied during post-processing, without modifying the model structure (e.g., layer of ME algorithm for parameter learning in [12]) or retraining. This allows for seamless integration with existing piecewise models, such as Vector-Switched GMP, and provides a lightweight solution to mitigate discontinuities in the inference phase, while maintaining a high level of accuracy. Therefore, the orthogonal interval approach is the methodology we will follow in this study.

Having established the theoretical framework, we now illustrate the practical implications using a well-known piecewise modeling technique.

### 2.2. Vector-Switched Generalized Memory Polynomial

The Vector-Switched Generalized Memory Polynomial (VS-GMP) model [4], as applied in [5], is used here to illustrate how discontinuities can arise in piecewise modeling. While this example focuses on VS-GMP, the issue is common to any method that combines sub-models. The insights and solutions discussed are broadly applicable.

The Generalized Memory Polynomial (GMP) is a well-established technique for modeling PA nonlinearities [15,16]. It combines the input into terms that capture different aspects of the amplifier’s behavior. For the sake of brevity, we will not describe the GMP equation, since it is a well-established method in the literature.

The input-power-dependent VS-GMP is a simple piecewise approach that splits the dataset into power regions, each modeled independently. The outputs from these sub-models are then stitched together into a single time series, as shown in Equation (12):

(12) $$y_{GMP-VS}=\left\{\begin{array}{cc} f_{1}\left(x\right) & \left|x\left(n\right)\right|\leq x_{1}\\ f_{2}\left(x\right) & x_{1}<\left|x\left(n\right)\right|\leq x_{2}\\ \vdots & \vdots \\ f_{i}\left(x\right) & |x\left(n\right)|>x_{i} \end{array}\right.$$

where |x(n)| is the absolute value of the input complex I/Q samples, $f_{i}\left(x\right)$ is the polynomial function representing each sub-model, and $x_{i}$ denotes the power threshold of each data partition.

Although Equation (12) uses the terminology of the case study, it is mathematically equivalent to the general piecewise formulation shown in Equation (11) using ${\mathrm{rect}}_{i}$ instead of ${\tilde{\mathrm{rect}}}_{i}$. Both describe how different sub-models are applied to different regions of the input signal.

The VS-GMP approach offers several advantages:

- Modularity: Each sub-model can be optimized independently, allowing for tailored complexity per region.
- Scalability: Additional regions can be added to improve accuracy in highly nonlinear zones.
- Implementation Simplicity: The switching mechanism is straightforward and computationally efficient.

However, a key drawback is the potential for discontinuities at the region boundaries. These arise when adjacent sub-models differ significantly in structure (e.g., polynomial order and memory depth), leading to abrupt changes in the predicted output. Such discontinuities can degrade the overall model performance, particularly in spectral domains, and may introduce artifacts in DPD applications. Therefore, in the next section we present the methodology used to characterize and correct these discontinuities.

## 3. Methodology for Characterizing Discontinuities and Compensation Proposal

### 3.1. Discontinuity Characterization

**Figure 4:**
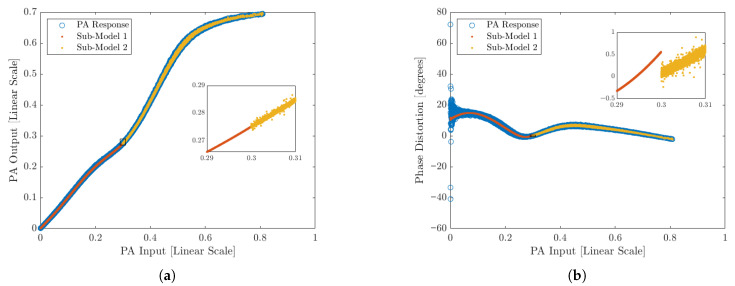
Example of discontinuity on a 20 MHz modulated signal. (**a**) Amplitude response with a spread discontinuity between sub-models. (**b**) Phase distortion with level and spread discontinuity between the sub-models.

Figure 4 shows the amplitude response and phase distortion of an A3M36SL039 Airfast Power Amplifier Module (NXP Semiconductors, Eindhoven, The Netherlands) [17] Doherty PA driven by a 4G Long-Term Evolution (LTE) waveform with 20 MHz bandwidth and a peak-to-average power ratio (PAPR) of 10.3 dB. To avoid bias during GMP coefficient estimation, the PA output was gain-compensated to match the input signal power:

(13) $$y_{\mathrm{comp}}=y\cdot \frac{{∥x∥}_{2}}{{∥y∥}_{2}},\;\mathrm{where}\;{∥z∥}_{2}=\sqrt{\sum_{n=1}^{N_{samples}}{\left|z\left[n\right]\right|}^{2}}.$$

where $N_{samples}$ is the number of samples of the time series z. All subsequent references to output data in this work correspond to $y_{\mathrm{comp}}$. Vectors are denoted using bold font.

This PA was arbitrarily modeled using two sub-models with the classic VS-GMP. The sub-model GMP parameters are purposely chosen to exacerbate the appearance of discontinuities around the knot ($x_{1}=0.3$) to aid the discussion.

Discontinuities are more evident in amplitude/phase distortion plots than in the time domain because the time series will lock the samples in place and it is hard to separate any eventual ringing due to discontinuity from the regular modeling error. Alternatively, the spectrum is expected to bury any components created due to discontinuities under the Noise Floor.

The proposed method to characterize discontinuities consists of analyzing variations in the statistical properties (conditional mean and variance) of the output of the model. Given the complex-valued samples

$$\left(x_{i},y_{i}\right)\in C^{2},\;i\in \left\{1,2,\ldots ,N\right\},$$

We now provide the empirical conditional mean E(Y∣X) and variance Var(Y∣X) for clarity. Here, *X* denotes the input magnitude and *Y* represents the aspect of the discontinuity being analyzed—either amplitude or phase:

- Amplitude analysis: Y=|y|;
- Phase analysis: $Y=∠\left(\frac{y}{x}\right)$, which corresponds to the AM-PM values.

We estimate the conditional quantities empirically using a local window W(x;ϵ)=[x−ϵ,x+ϵ] around *x* (0<ϵ≪1). We define the index set of data points within the interval W(x;ϵ) as follows:

(14) $$I_{x}=\left\{i\in \left\{1,2,\ldots ,N\right\}:\;|x_{i}|\;\in W\left(x;\epsilon \right)\right\}.$$

Let $n_{x}=\left|I_{x}\right|$ denote the cardinality of the index set $I_{x}$. The empirical conditional mean and variance are then defined as follows:

(15) $$\mu_{Y}\left(x\right)=E\left(Y|X=x\right)=\frac{1}{n_{x}}\sum_{i\in I_{x}}\left|y_{i}\right|$$

(16) $$\sigma_{Y}^{2}\left(x\right)=Var\left(Y|X=x\right)=\frac{1}{n_{x}}\sum_{i\in I_{x}}\left(\right|y_{i}{\left|-\mu_{Y}\left(x\right)\right)}^{2}.$$

Approximate derivatives of the empirical conditional mean and variance are then obtained using finite differences to analyze the variations:

(17) $$\begin{array}{cc} \Delta \mu_{k} & =\left|\Delta \left[\mu_{Y}\right]\left(x_{k}\right)\right|=\left|\mu_{Y}\left(x_{k}\right)-\mu_{Y}\left(x_{k-1}\right)\right|,\;\mathrm{and} \end{array}$$

(18) $$\begin{array}{cc} \Delta \sigma_{k} & =\left|\Delta \left[\sigma_{Y}\right]\left(x_{k}\right)\right|=\left|\sigma_{Y}\left(x_{k}\right)-\sigma_{Y}\left(x_{k-1}\right)\right|. \end{array}$$

**Figure 5:**
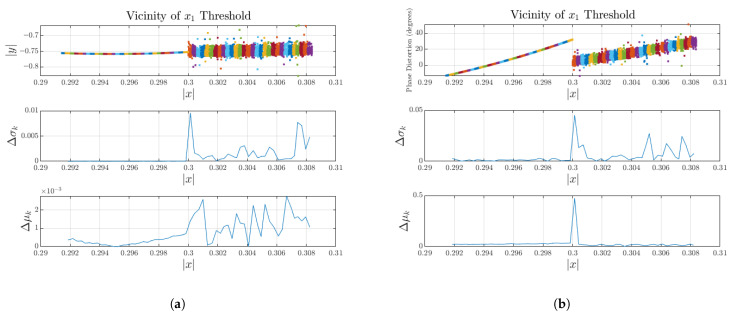
Conditional mean and conditional variance spike around the knot for the 20 MHz test case, demonstrating discontinuity. Local windows (W(x;ϵ)) are visualized with distinct colors to indicate the samples within each window. (**a**) Amplitude response discontinuity assessment. (**b**) Phase distortion discontinuity assessment.

Any significant discontinuity will appear as a spike between the two bins where a knot is located. Looking at the 20 MHz case shown in Figure 4, when the proposed statistical analysis is applied around the knot (Equations (15) and (16)), a clear variation can be observed in both the conditional mean and variance, as depicted in Figure 5.

It is important to emphasize that the values of $\Delta \mu_{k}$ and $\Delta \sigma_{k}$ should not be interpreted in absolute terms, but rather relative to the dataset under analysis. Power amplifier responses vary widely in absolute amplitude and phase due to differences in architecture, operating conditions, and signal characteristics. Therefore, discontinuities should be characterized by the presence of distinct spikes in $\Delta \mu_{k}$ and $\Delta \sigma_{k}$ at sub-model transition regions, indicating an abrupt change in the local mean or spread between adjacent bins.

The discontinuities of the conditional mean are attributed to a misalignment of polynomial order across models, whereas the conditional variance will have spikes in the presence of a memory depth mismatch.

This method offers a localized, quantitative assessment of continuity. Since the knot positions are known, there is no need to scan the entire dataset for discontinuities. Unlike time-domain derivative methods—prone to noise and memory effects—the statistical approach is robust and well-suited for real-world PA data with complex trajectories. Once discontinuities are identified, the next step is to apply a smoothing function that blends the outputs of adjacent models.

**Figure 6:**
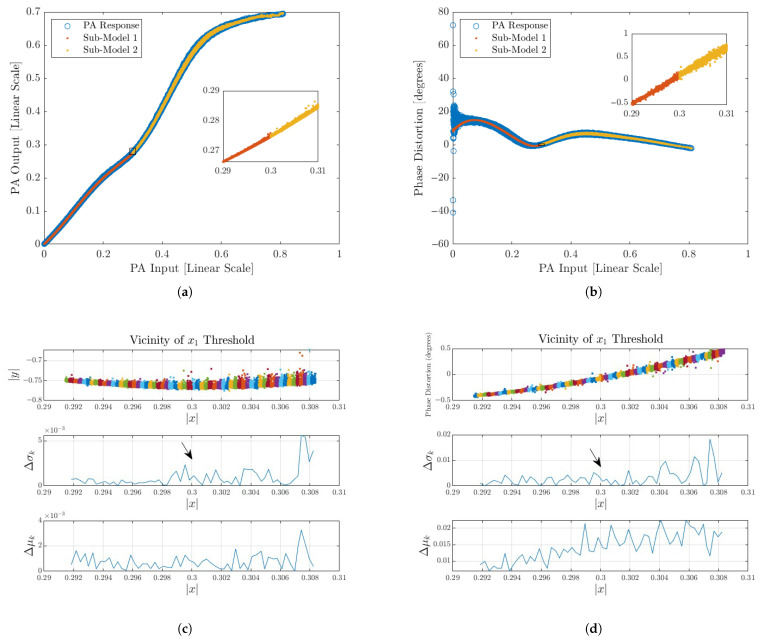
Depiction of the smoothed output data and the corresponding continuity assessment. The arrow denotes the spike location prior to processing; the spike is no longer visible afterward. Local windows (W(x;ϵ)) are visualized with distinct colors to indicate the samples within each window. (**a**) Input vs. output amplitude. (**b**) Phase distortion. (**c**) Amplitude discontinuity assessment. (**d**) Phase discontinuity assessment.

Applying a smoothing function at the model transition regions effectively reduces the peaks in $\Delta \mu_{k}$ and $\Delta \sigma_{k}$, resulting in a more continuous and coherent response, as illustrated in Figure 6. The smoothing approach employed in this case is detailed in the following section.

While various weighting functions can help reduce discontinuities in piecewise modeling, their selection involves a trade-off between implementation complexity and smoothness. In principle, any bell-shaped (i.e., symmetric) function can be employed. In this work, we adopt the raised cosine function [18] due to its simplicity and smooth roll-off characteristics, which make it well-suited for developing this proof of concept. A systematic study on the impact of different blending functions is left as future work.

### 3.2. Low-Complexity Smoothing with the Raised Cosine Weighting Function

The raised cosine weighting function (Equation (19)) is particularly advantageous for smoothing discontinuities between two polynomials due to its ability to effectively manage the transition between these segments. Therefore, it is defined as the overlapping basis function described in Equation (9).

(19) $${\tilde{\phi }}_{i}\left(x\right)=\left\{\begin{array}{cc} 1, & \left|x\right|\leq \frac{1-\beta }{2T}\\ \frac{1}{2}\left[1+\cos\left(\frac{\pi T}{\beta }\left[\left|x\right|-\frac{1-\beta }{2T}\right]\right)\right], & \frac{1-\beta }{2T}<\left|x\right|\leq \frac{1+\beta }{2T}\\ 0, & \mathrm{otherwise} \end{array}\right.$$

This weighting function is characterized by its roll-off factor, denoted as β, which controls the degree of smoothing. A higher β value results in a wider transition band, providing a smoother transition and reducing the abruptness of discontinuities. Conversely, a lower β value narrows the transition band, approaching the behavior of a pure rectangular function as β→0.

**Figure 7:**
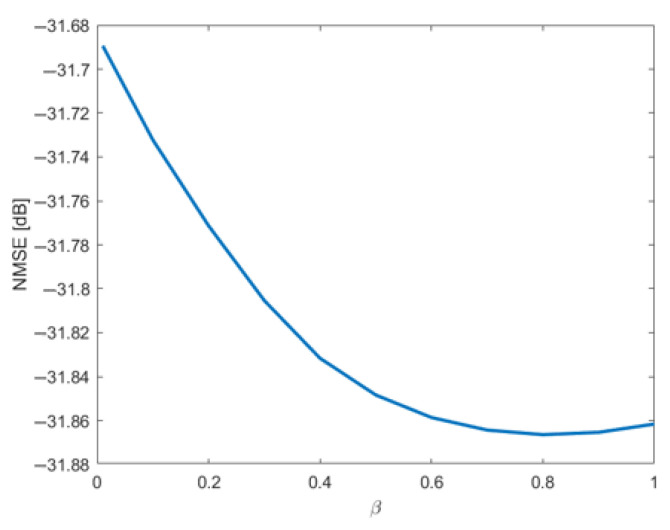
Parametric sweep to determine the optimal β.

It is important to note that the optimal β value is dataset-dependent, as it varies with the severity and nature of the discontinuities present in the modeled data. Therefore, the selection of β should ideally be performed for each new dataset. To determine the best value, we propose an NMSE-based parameter sweep, as illustrated in Figure 7.

Though the modeling performance is better around β=0.8, it is possible to observe that for this dataset the effect of β is limited to the absolute value of NMSE.

The raised cosine weighting function is applied exclusively at the transition regions between adjacent sub-models, ensuring that smoothing occurs precisely where discontinuities are most likely to arise. This process is performed during post-processing, allowing seamless integration with the modeled data without altering the original model coefficients.

This post-processing step completes the setup for the experimental validation. In the next section, we verify the appearance of discontinuities in VS-GMP with datasets extracted from real PAs and evaluate how the proposed smoothing technique affects the predicted output.

## 4. Experimentation and Results

To validate the proposed smoothing technique under diverse operating conditions, two power amplifiers (PAs) with distinct architectures were further characterized and modeled: the aforementioned NXP A3M36SL039 Doherty PA and the NXP BTS6201U Single-Stage PA [19]. This choice aims to demonstrate that discontinuities in piecewise polynomial models are not PA architecture-dependent and that the proposed method consistently improves NMSE across different PA types.

The Doherty PA was tested at its nominal center frequency of 3.6 GHz, while the Single-Stage PA was evaluated at 2.6 GHz. These frequencies correspond to typical 5G deployment bands ([20], Table 6.6.3.2-1) and were chosen to minimize gain and phase deviations at the edges of the operational bandwidth.

To assess the robustness of the smoothing method, the PAs were driven with New Radio (NR) and LTE modulated signals of varying bandwidths and PAPRs.

The Doherty PA was tested with three signals:

- 1× LTE 20 MHz at 10.3 dB PAPR;
- 1× NR 100 MHz at 10.3 dB PAPR;
- 2× NR 100 MHz aggregated carriers at 10.3 dB PAPR.

The Single-Stage PA was evaluated with

- 1× LTE 20 MHz at 7 dB PAPR;
- 1× NR 100 MHz at 7 dB PAPR;
- 1× NR 100 MHz at 11 dB PAPR.

These configurations allow us to explore different compression levels and memory effects. A higher bandwidth and PAPR typically increase modeling complexity and exacerbate conditional variance discontinuities. The aggregated carrier case for the Doherty PA was included to stress the model under realistic 5G scenarios with strong memory effects.

Each sub-model was constructed using a GMP structure tailored to its respective operating region. To balance accuracy and complexity, we employed the Pareto front—a widely used multi-objective optimization tool in PA modeling [21,22,23,24].

The methodology of Vector-Switched GMP proposed in [5] was employed to evaluate the presence of discontinuities in piecewise modeling and their impact on the predicted output within the current state of the art. This approach revealed issues with continuity. The optimization of knot locations using a grid search, as described in [5], resulted in placements at

- Doherty PA: $x_{1}=0.09$ | $x_{2}=0.17$ | $x_{3}=0.27$;
- Single Stage PA: $x_{1}=0.2$ | $x_{2}=0.4$.

The number of sub-models was also changed to test the continuity effect with different VS-GMP configurations.

To smooth the curve, ξ=0.05 was used in all partitions. This choice was made after a parameter sweep of ξ vs. final NMSE, similar to that in Figure 7.

**Figure 8:**
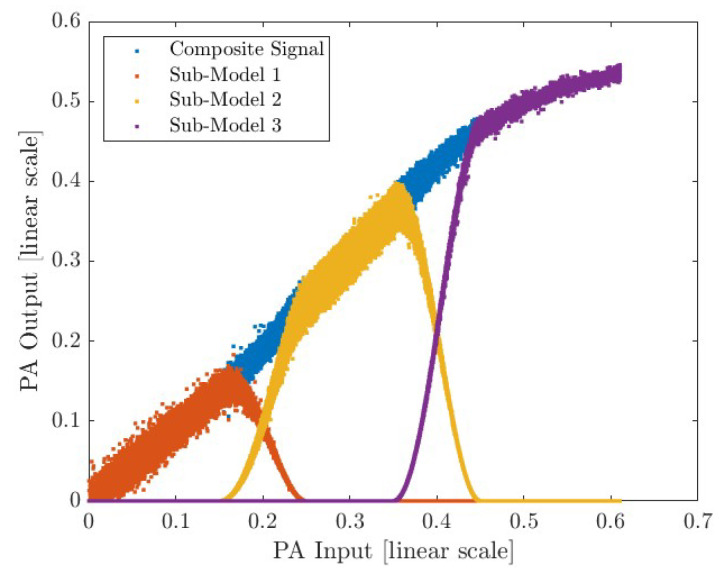
Final predicted output signal formed by the addition of the three smoothed sub-models.

As an example, the blended sub-models for the Single-Stage PA driven by an NR signal with 100 MHz bandwidth and a 7 dB PAPR are shown in Figure 8. It is possible to observe each partition after applying the blending function and its resulting smoothed composite.

**Figure 9:**
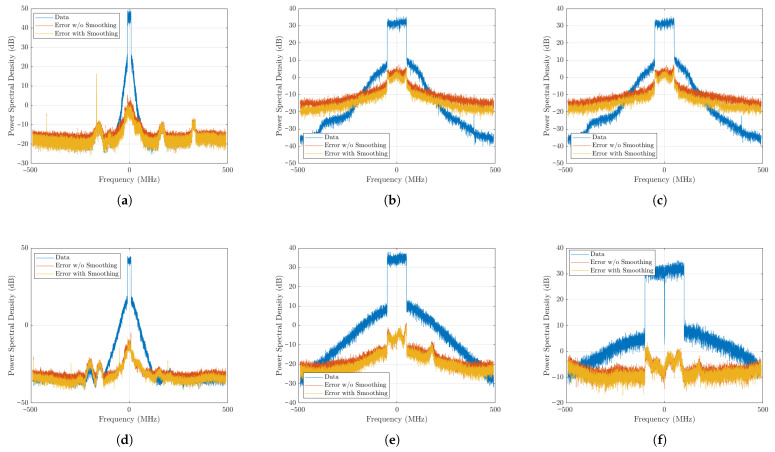
Predicted output error in frequency before and after smoothing for all test cases. (**a**) Single-Stage—1× LTE20 MHz. (**b**) Single-Stage—1× NR100 MHz and 7 dB PAPR. (**c**) Single-Stage—1× NR100 MHz and 11 dB PAPR. (**d**) Doherty—1× LTE20 MHz. (**e**) Doherty—1× NR100 MHz. (**f**) Doherty—2× NR100 MHz.

The results presented in Table 1 and Figure 9 confirm the effectiveness of the proposed smoothing technique across all tested scenarios. For every configuration, the application of the raised cosine weighting function reduced the spectral error, as evidenced by the error in the frequency-domain plots. This improvement was observed even in cases where the NMSE gain was modest, such as the 200 MHz scenario. Importantly, none of the test cases degraded the accuracy metrics of the model after the smoothing, which reinforces the robustness of the proposed approach.

The Single-Stage PA, used as a pre-driver with nominal operation at 17 dB back-off from P_1dB_, was tested in compression (5 dB back-off relative to P_1dB_) to better assess the impact of stronger nonlinearities on the appearance of discontinuities. This operating condition explains the higher NMSE observed compared to the Doherty PA.

As mentioned earlier, the impact of smoothing varies with model complexity and signal characteristics. In general, the proposed technique provides the largest NMSE improvements in configurations where the base model is less complex, such as the intentionally underfitted 100 MHz with 11 dB PAPR case. This behavior can be explained by the fact that underfitted models exhibit more pronounced discontinuities at the segment boundaries, which the smoothing function mitigates. These findings suggest that smoothing could be particularly beneficial in complexity-constrained scenarios, where the number of coefficients must be limited due to hardware or computational restrictions.

Another important observation is that the method remains effective under challenging conditions, such as high bandwidth, high PAPR, and aggregated carriers. Importantly, this performance gain is achieved without the need to retrain or re-estimate the model coefficients, which means that any given dataset with discontinuity could benefit from this modular approach during the inference phase, as a post hoc optimization technique.

## 5. Discussion and Conclusions

The proposed smoothing technique addresses a common yet often overlooked issue in polynomial piecewise modeling: the appearance of discontinuities at the boundaries between sub-models. Unlike previous approaches that require structural changes or retraining, this method operates entirely in post-processing, preserving the original model coefficients and enabling seamless integration into existing workflows. By applying a raised cosine weighting function at the transition regions, the predicted response becomes continuous and coherent, even when the sub-models differ significantly in polynomial order or memory depth.

The statistical characterization introduced here—based on localized variations in conditional mean and variance—offers a robust and interpretable way to assess continuity, avoiding the limitations of time-domain derivative methods. In the experimental validation, smoothing consistently reduced the spectral error across all test cases, as shown in Figure 9, with consistent NMSE improvement (typically between 1 dB and 3 dB) without any retraining needed. Importantly, no scenario was observed where smoothing degraded the accuracy metrics. This robustness suggests that the method can be safely applied as a post hoc enhancement without risk of performance loss.

The effect of smoothing was more pronounced in underfitted or complexity-constrained models, such as the 100 MHz, 11 dB PAPR case, where discontinuities were more severe around knot $x_{2}$, resulting in an approximate hundred-fold decrease in $\Delta \sigma_{x_{2}}$ and $\Delta \mu_{x_{2}}$ for both amplitude and phase. This indicates that the technique is particularly useful when the number of coefficients must be limited due to hardware or computational constraints. While the raised cosine function proved effective, future work could explore alternative blending functions such as Gaussian, bell curve, and sigmoid transitions to further optimize smoothness and adaptability.

While we show that discontinuities can arise from switching between different GMP polynomial compositions, it is also worth pointing out that different pruning strategies could lead to similar effects. Pruning changes the structure of the model, which can impact the smoothness across segments. With proper smoothing in place, it becomes feasible to mix different polynomial modeling techniques within a single segmented dataset—like combining GMP and pruned Volterra series [25]. This opens up the possibility of adapting the model locally to the behavior of each segment while keeping the overall response continuous. Additionally, the methodology is not limited to PA modeling; it can be applied to any discontinuous curve, making it a versatile tool for broader modeling applications in control systems, sensor calibration, or machine learning regression.

## Figures and Tables

**Table 1:** Comparison of NMSE and continuity metrics before and after applying the proposed smoothing technique across six PA model configurations.

						VS-GMP [5]				VS-GMP After Proposed Smoothing			
PA	PA Architecture	Center Freq. [GHz]	PAPR [dB]	Signal BW [MHz]	# GMP Coeff.	NMSE [dB]	Δμ/Δσ [Amp.|Phase] @ $x_{1}$	Δμ / Δσ [Amp.|Phase] @ $x_{2}$	Δμ / Δσ [Amp.|Phase] @ $x_{3}$	NMSE [dB]	Δμ / Δσ [Amp.|Phase] @ $x_{1}$	Δμ / Δσ [Amp.|Phase] @ $x_{2}$	Δμ / Δσ [Amp.|Phase] @ $x_{3}$
[17]	Doherty	3.6	10.3	20	78	−49.9	0.0006/0.004 | 0.083/0.03	0.003/0.003 | 0.04/0.06	0.004/0.007 | 0.06/0.02	−51.6	0.0002/0.001| 0.0004/0.03	0.0005/0.003 | 0.03/0.06	0.002/0.002 | 0.01/0.009
				100	90	−37.8	0.026/0.018 | 0.21/0.084	0.043/0.0042 | 0.37/0.1	0.04/0.03 | 0.20/0.12	−38.5	0.0006/0.008 | 0.04/0.1	0.027/0.007 | 0.02/0.02	0.001/0.02 | 0.03/0.04
				200	99	−31.0	0.038/0.024 | 0.32/0.069	0.050/0.037 | 0.52/0.35	0.07/0.1| 0.11/0.07	−31.9	0.01/0.02 | 0.08/0.01	0.009/0.007 | 0.02/0.15	0.007/0.1| 0.03/0.02
[19]	Single Stage	2.6	7.0	20	74	−38.8	0.04/0.01 | 0.02/0.01	0.013/0.005 | 0.002/0.008	Not applicable	−40.1	0.01/0.001| 0.002/0.005	0.002/0.005 | 0.001/0.005	Not applicable
			7.0	100	93	−27.4	0.054/0.37 | 0.02/0.06	0.056/0.0018 | 0.84/0.37	Not applicable	−29.8	0.02/0.06 | 0.10/0.37	0.004/0.0007 | 0.08/0.05	Not applicable
			11.0	100	70	−27.2	0.065/0.03| 0.22/0.085	0.11/0.02 | 1.2/0.74	Not applicable	−29.8	0.02/0.005| 0.002/0.05	0.006/0.03 | 0.03/0.11	Not applicable

## Data Availability

The dataset acquired for this study used a full test setup with a commercial PA [19]. It is not readily available because it has been acquired using proprietary equipments and softwares from NXP Semiconductors. Access to the raw data may be granted upon reasonable request and subject to NXP Semiconductors’ approval. Requests to access the datasets should be directed to the corresponding author.

## Abbreviations

The following abbreviations are used in this manuscript:

PA	Power Amplifier
RF	Radio Frequency
DPD	Digital Predistortion
GMP	Generalized Memory Polynomial
VS-GMP	Vector-Switched Generalized Memory Polynomial
AMAM	Amplitude-to-Amplitude Distortion
AMPM	Amplitude-to-Phase Distortion
NR	New Radio
I/Q	In-Phase and Quadrature Components
NMSE	Normalized Mean-Square Error
BW	Bandwidth
PAPR	Peak-to-Average Power Ratio
